# The National Medical Union (Official)

**Published:** 1914-03-28

**Authors:** 


### THE NATIONAL MEDICAL UNION (Official).

Offices: 346 Strand. Tel.: City 8444.

### THE RESOLUTIONS ADOPTED LAST SATURDAY.

A meeting of the Provisional Council was held on Saturday, March 21, at the offices of the Union, to deal with the subject of policy. The importance of the meeting was marked by the presence of the Honorary Presidents, Mr. G. A. Wright and Professor Russell. The meeting was a prolonged one, lasting from 4 p.m. till after 9 p.m. The ground covered by the resolutions printed in The Hospital of March 14 was satisfactorily cleared, but, owing to pressure of business, it has been impossible to issue a complete report of this meeting this week, but it is hoped that this will be published in full in the next issue of The Hospital. Meanwhile, it should be stated that the following resolutions were adopted by the Council with various modifications which will be detailed next week:—

Edinburgh.—Resolutions Nos. 2 and 3.

Crystal Palace.—Resolutions Nos. 3 and 4. In connection with No. 3 the resolution runs: "That the National Medical Union is not opposed to the principle of Voluntary Health Insurance," etc., while to No. 4 are added the words "under the present arrangements."

Wandsworth.—Resolutions Nos. 1 and 2, the latter covered by No. 2 of Hampstead. Resolution No. 3 amended as follows: "Repeal of the Medical Benefit Sections or such drastic amendments as would bring these sections into harmony with the principle of freedom on the part of the profession and public as laid down in the policy of the Union." Resolution No. 4 was referred to a sub-committee.

Hampstead.—Resolution No. 2, first paragraph, with an addition. Resolutions Nos. 3 and 4, Resolution No. 5, first three lines.

In the course of the past week the results of the nominations for the election of the Local Medical Committee, proposed by the Metropolitan Counties Branch of the British Medical Association, were published, showing the remarkable abstinence on the part of non-panel practitioners from allowing themselves to be nominated for this election. Attention is directed to the publication of this list in the *Lancet*, March 21, page 864, where in a parallel column will be found an exposition of the situation, together with the ground upon which the London Organisation Committee of the National Medical Union took action. At the meeting on Saturday the Council heartily approved of the action which this committee had taken in this matter.

### THE PROFESSION OF MEDICINE.

### Leicester Union of Medical Practitioners.

The second annual meeting of the Union was held at the Public Medical Service Buildings, Leicester, on Friday, February 27. Dr. Wallace Henry was elected president, Dr. J. B. Pike vice-president, and Dr. A. V. Clarke treasurer. The Secretary reported that the membership had increased during the year by 105.

Arrangements have been made to enable members to obtain, without any charge, legal advice on matters connected with the relations between themselves and their patients, to have debts collected, and to effect insurances on extremely favourable terms. The Treasurer reported that there was a balance in hand of £132 18s. The committee was authorised to consider the desirability of federating with the National Medical Guild.

The meeting was followed by the annual meeting of the Leicestershire and Rutland Public Medical Service, which is managed by the Union. Reports were received from the various sub-divisions of the Service, which showed that there were over eighty thousand uninsured persons subscribing members; that the year's work and financial results were most satisfactory. Suggestions for further improving the work of the Service were referred to the committee.

### Some Miscarriages of Medical Benefit.

Every week, almost every day, brings to light some fresh instance of hardship, suffering, and even actual death under the provisions of the Insurance Act medical benefit as at present administered. Many of these cases are clearly and indisputably the result of carelessness, if not of gross negligence, on the part of a few members of the medical profession. But there are two aspects of these miscarriages of benefit which cannot be altogether passed over with the philosophical reflection that accidents will happen in the best regulated legislation.

First, to what extent can these unfortunate incidents be ascribed to the panel system and the general policy of the Government in enforcing that system in the way they did? Secondly, what action are the responsible authorities of the medical profession taking to deal with cases of obvious negligence on the part of practitioners? These two questions are totally distinct, in that one refers to a matter of political economy, and the other is a purely domestic question for the General Medical Council. But each case that arises prompts inquiry into both questions, so that there is an indirect connection in this way between them.

Let us consider briefly one of the worst of these cases, in which a Coroner's jury recently censured, with the full approval of an experienced London Coroner, the panel practitioner in charge of the case. A foreman sewerman felt ill and consulted his panel doctor—Dr. D. J. F. Bennett—and was given a bottle of medicine, but was not examined. Feeling worse instead of better, he went again; again he was not examined, but was told he was suffering from rheumatism in the chest, a label which is in itself ridiculous. Shortly afterwards, while still at work, the man fell down unconscious and was taken to hospital in a taxicab; he died before the hospital was reached. The *post-mortem* examination revealed extensive tuberculosis of the lungs, with cavities in both apexes an inch and a half across. It is incontestable that this was an instance of the grossest negligence on the part of the panel doctor, who did not put in an appearance at the inquest, but wrote to the Coroner admitting he had never examined the patient.

An official of the National Health Insurance Committee, it is stated, was present in court to watch the proceedings, and it remains to be seen what action the Committee will take when they have considered their official's report. That, however, is not enough; the Committee may possibly strike his name off the panel; but this is the limit of their disciplinary powers. We consider that this case, and others of the same sort, demand investigation by the General Medical Council. If the statements made at the inquest, accepted and acted upon by the Coroner and the jury, are proved to be true, severe and rigorous action is absolutely essential for the preservation of the fair fame of the medical profession. The *Sheffield Independent* would "like to see the doctor put on his trial for manslaughter who diagnosed tuberculosis as rheumatism, and if he were sent to gaol there would be the elements of justice in such a procedure which were conspicuous by their absence when a driver was sent to gaol for overrunning a signal." In law, presumably, it would be exceedingly difficult to found a charge of manslaughter on the conduct of this practitioner. But when newspaper editors commit themselves to language as strong as this, it is evident that public feeling is considerably stirred. At the very least, we think, the published facts of the case would justify a charge of "infamous conduct in a professional respect," the penalty for which is, or may be, erasure from the Medical Register.

There are, as is well known, many practitioners up and down the kingdom who have accepted so many thousands of insured persons upon their panel lists that it is humanly impossible to give proper attention to that proportion of their panel patients who are in need of it at any given time. Such practitioners may have the fullest intentions of examining carefully every patient who presents himself, but it is absolutely certain that they cannot carry them out. Doubtless an experienced clinician can rely on his trained eye to pick out from the crowd in the surgery those who are most ill and most in need of complete physical examination. But that is not the method which any conscientious man pursues, because it is a mathematical certainty that sooner or later (probably sooner) mistakes will be made, and wrong diagnoses arrived at. It is not the method pursued, for example, in the out-patient rooms of the voluntary hospitals, where the medical officers are of much higher professional status and experience than panel practitioners can ever hope to be. It follows that panel doctors with over-swollen lists are inevitably guilty of inefficient, and therefore inexcusable, treatment of their panel patients, though happily most of them avoid such horrible tragedies as the above. And it also follows that a portion of the blame for this state of affairs attaches to those in charge of the administration of the Act, who have not merely permitted, but have actually encouraged, panellers to accept thousands of names in excess of the number to which they can adequately attend.

### The "Family Encyclopædia."

It might, however, conceivably be urged in restraint of the conclusions at which we have arrived in the preceding paragraphs, that the General Medical Council can hardly be too severe upon humble panellers, as long as it turns a blind eye to offences against professional ethics committed by those who consider themselves the *élite* of the profession. In our last issue (p. 685) some pertinent questions were put by a correspondent who commented in caustic terms upon the association of a number of eminent consultants with the advertisements of a new "Family Encyclopædia of Medicine."

To that argument it is easy enough to reply that two wrongs do not make a right; and that because some highly placed offenders against professional ethics are unpunished, that is no reason why all wrong-doers should be. Logically, the answer is complete; but, humanly speaking, it fails to carry conviction. Obviously, it should be impossible for anyone to maintain with the slightest show of reason that disciplinary action is not taken against offenders who happen to be fashionable consultants, whereas it is taken against lesser lights for similar offences. We do not consider, for the moment, whether or not such disciplinary action could or ought to be taken against the thirty-two doctors—or any of them—whose names were so successfully advertised by the Harmsworth publishing house a month or so ago. We merely put forward the proposition that the General Medical Council should never, by its actions, give anyone the slightest ground for questioning its absolute impartiality in matters of this sort.

Turning more particularly to the letter of our correspondent last week, and reviewing the disclaimers put forward by the thirty-two, both individually and collectively, we feel obliged to say that these gentlemen have not shown a sufficient sense of their error, and of their responsibility to the profession for its consequences, in their published statements. Many of them, undoubtedly, were innocent of any wrongful intent, and genuinely annoyed when they found to what use their names and their co-operation had been put; but even these consultants should have been much more circumspect and less childlike in their innocence of the ways of the world. Others, we shrewdly suspect, have even less excuse for their indiscretion. And none of them have yet done enough, or nearly enough, towards repairing the damage done by their default to the good name of the medical profession and the cause of professional honour.

